# Dyeing Property and Adsorption Kinetics of Reactive Dyes for Cotton Textiles in Salt-Free Non-Aqueous Dyeing Systems

**DOI:** 10.3390/polym10091030

**Published:** 2018-09-15

**Authors:** Jiping Wang, Yuanyuan Gao, Lei Zhu, Xiaomin Gu, Huashu Dou, Liujun Pei

**Affiliations:** 1 Engineering Research Center for Eco-Dyeing and Finishing of Textiles, Zhejiang Sci-Tech University, Hangzhou 310018, Zhejiang, China; jpwang@zstu.edu.cn (J.W.); aloysyy@163.com (Y.G.); lzhuzj@163.com (L.Z.); abbiexiaowang@hotmail.com (X.G.); 2 National Base for International Science & Technology Cooperation in Textiles and Consumer-Goods Chemistry, Zhejiang Sci-Tech University, Hangzhou 310018, Zhejiang, China; 3 Key Laboratory of Advanced Textile Materials & Manufacturing Technology, Ministry of Education, Zhejiang Sci-Tech University, Hangzhou 310018, Zhejiang, China; 4 Key Laboratory of Fluid Transmission Technology of Zhejiang Province, Zhejiang Sci-Tech University, Hangzhou 310018, Zhejiang, China; huashudou@zstu.edu.cn

**Keywords:** non-aqueous medium dyeing, salt-free reactive dyeing, cotton textile, reactive dye, surface tension, adsorption

## Abstract

In recent years, new concepts in textile dyeing technology have been investigated which aim to decrease the use of chemicals and the emission of water. In this work, dyeing of cotton textiles with reactive dyes has been investigated in a silicone non-aqueous dyeing system. Compared with conventional aqueous dyeing, almost 100% of reactive dyes can be adsorbed on cotton textiles without using any salts in non-aqueous dyeing systems, and the fixation of dye is also higher (80%~90% for non-aqueous dyeing vs. 40%~50% for traditional dyeing). The pseudo-second-order kinetic model can best describe the adsorption and equilibrium of reactive dyes in the non-aqueous dyeing systems as well as in the traditional water dyeing system. In the non-aqueous dyeing systems, the adsorption equilibrium of reactive dyes can be reached quickly. Particularly in the siloxane non-aqueous dyeing system, the adsorption equilibrium time of reactive dye is only 5–10 min at 25 °C, whereas more time is needed at 60 °C in the water dyeing system. The surface tension of non-aqueous media influences the adsorption rate of dye. The lower the surface tension, the faster the adsorption rate of reactive dye, and the higher the final uptake of dye. As a result, non-aqueous dyeing technology provides an innovative approach to increase dye uptake under a low dyeing temperature, in addition to making large water savings.

## 1. Introduction

In recent years, tremendous efforts have been focused on saving water and energy in the textile industry due to environmental pressure [1,2,3,4]. Some new dyeing methods [5,6,7,8] have been developed to reduce the use of salts and to save water during the dyeing process; for example, supercritical fluid dyeing, solvent dyeing, reverse micelle dyeing, non-aqueous dyeing, ionic liquid dyeing etc. Unfortunately, natural fibers cannot be effectively dyed with conventional water-soluble dyes in normal supercritical fluid [4,5]. Solvent dyeing and reverse micelle dyeing are often carried out using hydrocarbon solvents which are not environmentally friendly, such as hexane, cyclohexane and n-heptane, as continuous phase media [9,10]. Non-aqueous dyeing is a green and environmentally friendly dyeing technology which uses non-polar media to substitute for water [11,12]. Moreover, Yang [9] and Fu [13] believe that non-aqueous dyeing has been the most successful attempted substitute for traditional water-based dyeing in the textile industry. Non-aqueous dyeing media cannot dissolve water-soluble dye but can transport material and transfer energy. A major advantage of non-aqueous dyeing technology is that little water is needed because dissolving the dye and other chemical agents, and fiber swelling require only a small amount of aqueous solution [14,15,16]. Furthermore, all the aqueous solution can be completely absorbed by the cotton textile without using any accelerating salts during the dyeing process. As for the re-usability of non-aqueous media, after a short period of static separation non-aqueous media can be directly used for the next dyeing. Non-aqueous media which is adsorbed on the surface of cotton textile can be washed down using surfactants and reused by flotation.

**Figure 1:**
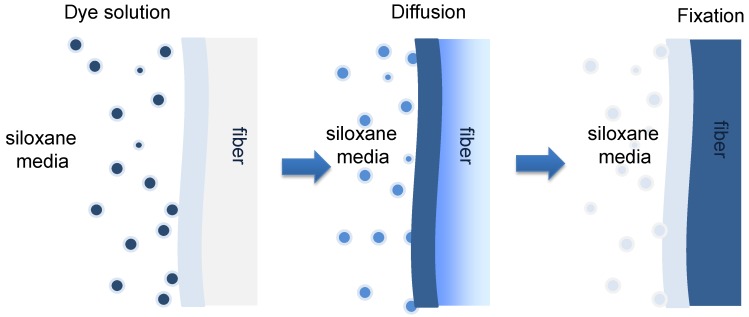
Schematic diagram of reactive dye in siloxane non-aqueous dyeing system.

In our previous investigations [15,17,18,19], a non-aqueous dyeing system was favorably prepared using siloxane non-aqueous medium as the dyeing medium. Siloxane non-aqueous medium is a clear, odorless, colorless, and non-oily cyclic siloxane fluid, which is widely used in consumer and industrial applications. Research has demonstrated that siloxane non-aqueous medium is safe to both human health and the environment [20,21,22]. In recent years, siloxane non-aqueous medium has been applied widely in dry cleaning as a new medium [23]. Now, we have used this non-aqueous medium to prepare a reactive dye/siloxane emulsion dyeing system for dyeing cotton textiles. As shown in Figure 1, with the aid of a surfactant and *co*-surfactant, the dye solution can be evenly emulsified in siloxane non-aqueous medium. Since reactive dye is totally incompatible with siloxane, there is a strong affinity between the fiber and the dye, with the result that nearly 100% of reactive dye can diffuse to the surface of the cotton textile under mechanical force. Therefore, the final uptake of reactive dye is higher than that of a conventional water dyeing base, without using any inorganic salts in the non-aqueous dyeing base. Furthermore, the fixation of dye is also higher than in the water bath. As a result, a lot of reactive dyes can bond with cotton textiles in non-aqueous dyeing systems. However, information about the adsorption mechanism of reactive dye solution in the siloxane non-aqueous dyeing system is limited; in particular, comprehensive information related to the effect of the medium on the diffusion of reactive dye and adsorption models have not been systematically studied.

To study the influence of non-aqueous media surface tension on the adsorption of reactive dye, vinyl sulphone reactive dye (blue 19) and a combination of cyanuric chloride and vinyl sulphone reactive dye (red 195) were selected. The influences of dyeing media and surface tension of non-aqueous media on the adsorption kinetics of reactive dye were investigated with a model on the adsorption kinetics of reactive dye. In addition, the influence of surface tension on reactive dyeing level property was systematically discussed.

## 2. Materials and Methods

### 2.1. Materials

Cotton woven fabric (130.5 g/m^2^, yarn count: 42S × 42S, yarn density: 148 × 285) was dyed with reactive dye which was obtained from Haitong Printing&Dyeing Co., Ltd, (Hangzhou, China). Siloxane non-aqueous medium (purity>98%) was purchased from Wynca. Paraffin (Jiande, China), C_8_H_18_ (isooctane) and M-oil (white oil) withpurity above 99.7% were purchased from Hangzhou Mick Chemical Co., Ltd (Hangzhou, China) The parameters of different dyeing media are shown in Table 1.

**Figure 2:**
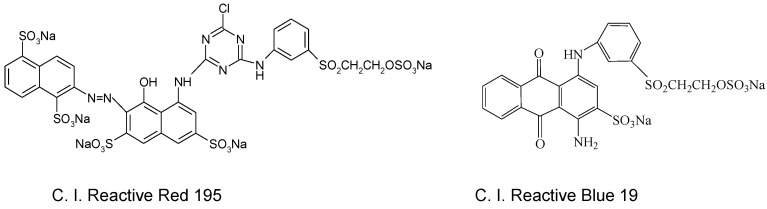
Molecular structure of C. I. (Color Index) Reactive Red 195 and C. I. Reactive Blue 19.

Reactive dyes (red 195 and blue 19) were purchased from Aladdin Reagent (Shanghai, China). The molecular structures of these dyes are shown in Figure 2. Non-ionic surfactant (AEO-3, fatty alcohol polyoxyethylene ether) was obtained from Jiangsu Haian Petrochemical Plant (Haian, China). *n*-octanol (A.R.) was purchased from Aladdin Reagent (Shanghai, China). Sodium carbonate (Na_2_CO_3_, A.R.) and sodium chloride (NaCl, A.R.) were purchased from Cangzhou Haolong Chemical Products Co., Ltd (Cangzhou, China).

### 2.2. Determination of Surface Tension

A Krüss GmbH DAS20 using the captive drop method was used to measure the surface tension of different non-aqueous media. In the testing conditions, the needle diameter was 1.820 mm and flow rate was 60.2 mm/min.

### 2.3. Cotton Textile Dyeing

#### 2.3.1. Non-Aqueous System

5.0 g cotton fabric was dyed with reactive dye (2%, o.w.f.) in 100 mL (mililitro) non-aqueous medium (liquor ratio = 20:1) using a dyeing machine (Sanjing Technology Company, Hangzhou, China). First, 2% (o.w.f.) of reactive dyewas added to 130% (o.w.f) of water. After stirring for 5 min, 7.5% (o.w.f) of sodium carbonate was added to reactive dye solution and stirred for another 5 min. Finally, the dye solution obtained in the previous step was added into 100 mL of non-aqueous medium. After adding the cotton fabric, dyeing began at a lower temperature (25 °C) for 15 min, and then the temperature was increased to 70 °C for 30 min, with a heating rate of 2°C/min. After dyeing, the dyed fabric was washed in a soap solution containing 2.0 g/L sodium carbonate and 2.0 g/L standard detergent for 10 min, rinsed again with warm (50 °C) water and cold water, and finally dried at ambient temperature.

#### 2.3.2. Aqueous Dyeing

For comparison with the conventional water-base dyeing, the same cotton fabric (5 g) was dyed with reactive dye (2%, o.w.f) in water base at a liquor ratio of 20:1. The amounts of NaCl and Na_2_CO_3_ were 40 and 15 g/L, respectively. First, reactive dye and the cotton fabric were added into 100 mL of water at room temperature, and then the temperature was increased to 60 °C. After 10 min, half of the NaCl was added into the dye bath, and the rest of the NaCl was added in the next 10 min. After adsorption (30 min), the temperature was increased to 70 °C at 2 °C/min, then the Na_2_CO_3_ was added into the dye bath and left for 30 min. After fixation, the dyed fabric samples were soaped and rinsed.

### 2.4. Determination of Adsorption Rate

The dyeing rate was monitored using the dye concentration. Small samples (1 mL) were taken at different times during the dyeing process. The concentration of dye was determined with a UV-vis spectrometer (Lambda 35, Perkin Elmer, Waltham, MA, USA).

Prior to dyeing, standard solutions of the two dyes were made and the absorbance was measured from 380~680nm wavelengths; therefore, a plot of absorbance versus wavelength could be obtained. Maximum adsorption wavelength of the dye was determined with a UV-vis spectrometer (C.I. Reactive Red 195 in 550 nm; C.I. Reactive Blue 19 in 596 nm). Calibration curves of absorbance vs. dye concentration (Beer-Lambert law) were performed separately for the two dyes. The calibration plot revealed a linear relationship in the range of dye concentration from 0 to 0.19 g/L. Therefore, it was necessary to dilute the sample with a higher concentration.

For the non-aqueous dyeing system, the dye solution (1 mL) was diluted with 9 mL of ethyl alcohol. For the traditional system, distilled water was used for dilution. Samples of dye solutions were taken at different times (0, 1, 3, 5, 10, 15, 20, 25, 30, 40, 50, 60 and 70 min) for UV measurements.

### 2.5. Dyeing Evaluations

#### 2.5.1. Color Depth of Dyed Fabric

The color strength of dyed fabric samples were measured with a Datacolor SF600X spectrophotometer (Datacolor, Lawrenceville, NJ, USA), then the K/S values were evaluated at λ_max_ using the Kubelka-Munk equation:

(1) $$\frac{K}{S}=\frac{{\left(1-R\right)}^{2}}{2R}\;$$

where *R* is the reflectance of the dyed fabric sample at the λ_max_ absorption and *K* and *S* are the absorption and scattering coefficients, respectively.

#### 2.5.2. Dye Uptake of Reactive Dye

The final uptake of reactive dye was calculated by the following equation. The amount of dye in the dyeing residues solution was determined using a UV-vis spectrometer.

Sorption% = (1 − *C*_1_*V*_1_/*C*_0_*V*_0_) × 100% (2)

where sorption refers to the dye uptake, *C*_0_ and *C*_1_ refer to the concentration (g/L) of initial dye solution and dyeing residues, respectively; and *V*_0_ and *V*_1_ refer to the volume (mL) of initial dye solution and dyeing residues, respectively.

#### 2.5.3. Fixation Rate of Reactive Dye

Consideration of the percentage of fiber-reactive dyes is necessary to evaluate the dye fixation during the non-aqueous dyeing systems and in the traditional water dyeing system. The fixation ratio of reactive dye was calculated based on the following equation:

(3) $$T=(1-\frac{C_{1}V_{1}+C_{2}V_{2}}{C_{0}V_{0}})\times 100\%\;$$

where *T* refers to the fixation rate of reactive dye, and *C*_0_, *C*_1_, *C*_2_ refer to the concentration (g/L) of the initial dye solution, dyeing residues and soaping solution, respectively. *V*_0_, *V*_1_, and *V*_2_ refer to the volume (mL) of the initial dye solution, dyeing residues and soaping solution, respectively.

#### 2.5.4. Level Dyeing Property

Twelve points on the fabric surface were randomly selected, and the K/S values of these points were tested with a Datacolor SF600X spectrophotometer (Datacolor, Lawrenceville, NJ, USA). The level dyeing property was calculated using the following equation, where the lower the value of *S_γ_*(*λ*), the better the level dyeing property:

(4) $$S_{\gamma }(\lambda )=\sqrt{\frac{\sum_{i=1}^{n}{\left[\frac{{\left(K/S\right)}_{i\lambda }}{\bar{{\left(K/S\right)}_{i\lambda }}}-1\right]}^{2}}{n-1}}$$

## 3. Results and Discussion

### 3.1. Comparisons between the Dyeing Property of Reactive Dye in Different Non-Aqueous Dyeing Systems and a Traditional Water Dyeing System

**Figure 3:**
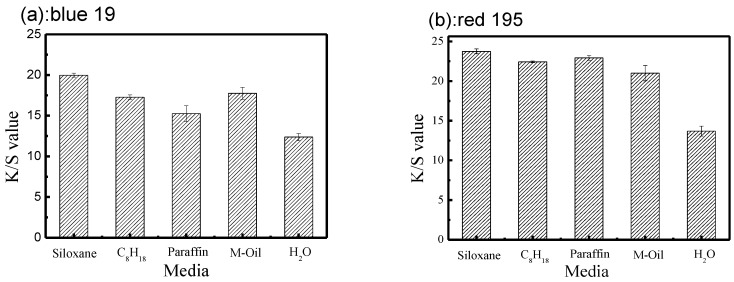
Color depth of fabric in different non-aqueous emulsion systems and traditional water base: (**a**) C. I. Reactive Blue 19; (**b**) C. I. Reactive Red 195.

Four different non-aqueous dyeing media were selected to study their influence on reactive dyeing for cotton fabric, and the color depths of the dyed fabric are plotted in Figure 3. Dyeing in a water base was also included for comparison. In the traditional water dyeing system, the color depth of cotton fabrics (*K*/*S* value) dyed with reactive blue 19 and red 195 were 12 and 13, respectively; while in non-aqueous media, the color depth of dyed cotton fabric samples dyed with reactive blue 19 and reactive red 19 were above 15 and 21, respectively.

Comparing the different dyes, the color depth of the cotton fabric dyed with reactive 195 was deeper than that of the fabric dyed with reactive blue 19. This was mainly because the reactive red 195 molecular structure contains two different active groups; namely, the homogeneous chlorotriazine and vinyl sulfone active groups, with the result that the reaction between fiber and dye was higher than that of a single functional group (blue 19) [24,25,26]. Furthermore, the molar extinction coefficients of dye might be a reasonable guide to the color depth of dyed fabric [27]. Reactive red 195 may absorb more light and reflect less light. Therefore, the color depth of fabric after dyeing with reactive red 195 was significantly deeper than that of cotton fabric dyed with reactive blue 19.

**Figure 4:**
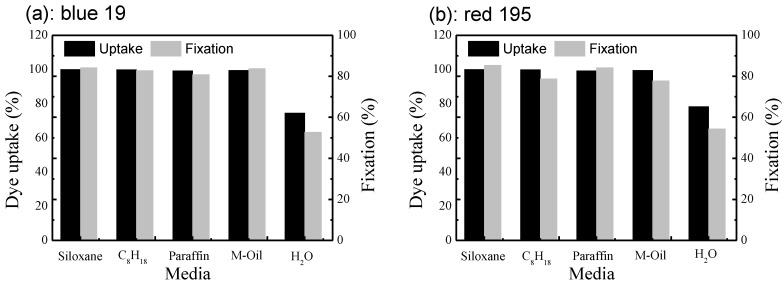
Uptake and fixation of reactive dye in different non-aqueous emulsion systems and traditional water base: (**a**) C. I. Reactive Blue 19; (**b**) C. I. Reactive Red 195.

As shown in Figure 4, the uptake of reactive dye in non-aqueous media was above 95%, indicating that almost all of the dye could diffuse to the cotton fabric without adding any electrolyte at 25 °C during dyeing. However, in the conventional aqueous dyeing system, the final uptake of these two reactive dyes was only 70%~75% when using an accelerating agent (sodium chloride) at 60 °C. Considering removal of these salts is a major challenge for the treatment of dyeing wastewater [25]; however, there is a little wastewater after dyeing in the non-aqueous media dyeing systems. Therefore, non-aqueous dyeing systems are green and recyclable dyeing systems.

The effect of the non-aqueous media on the fixation of the reactive dye was also investigated, as shown in Figure 4. The fixation rates for the non-aqueous dyeing systems were higher than for the traditional process (80%~90% for siloxane non-aqueous dyeing system vs. 40%~50% for traditional). Fixation is also affected by the sorption of dye, dyeing time, etc. [10,28]. The apparent sorption of reactive dye was over 95% in non-aqueous dyeing systems, indicating that a lot of the dyes could react with cotton fabrics. Furthermore, the dyeing temperature is lower (25 °C in non-aqueous media vs. 60 °C in water base), and the hydrolysis rate of reactive dye in non-aqueous dyeing systems is slower than that in the traditional water base [15,16]. As a result, a lot of reactive dyes can bond with cotton textiles, and a higher fixation rate can be obtained in the non-aqueous dyeing systems [29,30]. As can be seen, this dyeing technology can solve some of major problems of a traditional water bath, such as difficult wastewater treatment due to the high residue amounts of dye and electrolytes, low dye efficiency, etc.

### 3.2. Dye Adsorption in Different Non-Aqueous Dyeing Systems

The degree of dye adsorption (*q_t_*) can be calculated using the following equation:

(5) $$q_{t}=\frac{1000\times (C_{0}-C_{t})}{W}V\;$$

where *q_t_* (mg/g) refers to the mass of dye adsorbed on cotton fabric at given times during the dyeing process, *C*_0_ refers to the initial dye concentration (g/L) in the dye bath, *C_t_* refers to the residue dye concentration (g/L) at given times, *V* is the volume of dye solution (mL), and *W* is the mass of cotton fabric (g).

**Figure 5:**
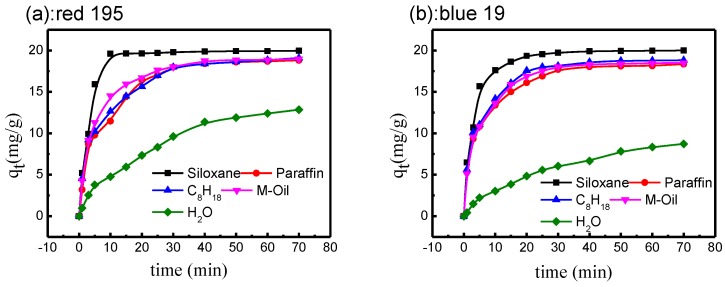
Adsorption of reactive dye in different non-aqueous dyeing systems and traditional water base: (**a**) C. I. Reactive Red 195; (**b**) C. I. Reactive Blue 19.

As shown in Figure 5, the fastest equilibrium was observed at 25 °C in the siloxane non-aqueous dyeing system. In other non-aqueous dyeing systems, adsorption equilibrium was established within 20–30 min. This is mainly because the surface tension of non-aqueous media is low (18.13 mN/m), which makes them highly hydrophobic, and reactive dye solution is easily adsorbed on the cotton fabric surface under mechanical force [31,32]. Therefore, salt-free adsorption could be realized by non-aqueous media dyeing.

In addition to the faster equilibrium, the highest uptake of dye (20 mg/g) was achieved after dyeing for 10 min in the siloxane non-aqueous dyeing system. For other non-aqueous dyeing media, the uptake of dye was also above 94%, which was significantly higher than that of the traditional water base. The distribution of reactive dye solution between the dyeing bath and the cotton fabric depends on its relative solubility in the dyeing media and fabric. Since reactive dye solution is totally incompatible with non-aqueous media, there is a strong affinity between fiber and dye, with the result that nearly 100% of reactive dye can diffuse to the surface of cotton textile under mechanical force [18,33].

### 3.3. Dye Adsorption Kinetics

#### 3.3.1. Fitting of Pseudo-First-Order Kinetic Model

In order to assess the adsorption performance of reactive dye in different non-aqueous dyeing systems, the adsorption dynamics of the reactive dye were evaluated using Lagergren equations [34,35,36,37].

(6) $$\ln(q_{e}-q_{t})=\ln q_{e}-k_{1}t\;$$

where *q_e_* (mg/g) and *q_t_* (mg/g) denote the masses of dye adsorbed on cotton fabric at equilibrium sate and at given times *t* (min) respectively, and *k*_1_ refers to the rate constant of the pseudo-first-order kinetic model.

According to Equation (6), *R*^2^ (correlation coefficient) was calculated using plots of ln(*q_e_* − *q_t_*) versus adsorption time (*t*).

**Figure 6:**
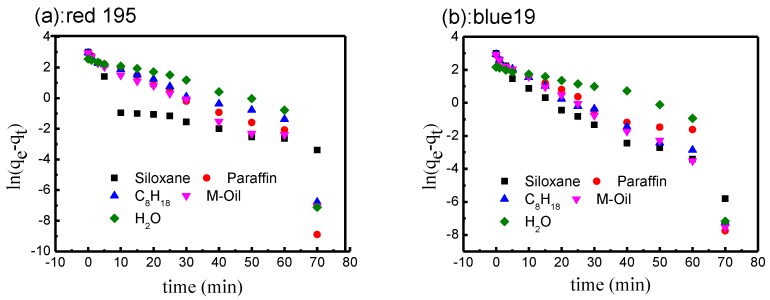
Kinetics of pseudo-first-order model for adsorption of reactive dyes: (**a**) red 195; (**b**) blue 19.

The fitted line plots of equations in non-aqueous dyeing systems and conventional water are shown in Figure 6. Table 2 shows the values of *R*^2^ for cotton fabric dyed with red 195 and blue 19. Both non-aqueous dyeing systems and the aqueous system showed a low correlation coefficient, indicating that the pseudo-first-order kinetic model did not fit well for the adsorption or reactive dye in different non-aqueous dyeing systems.

#### 3.3.2. Fitting of Pseudo-Second-Order Kinetic Model

The pseudo-second-order kinetic model is expressed as the following equation [38,39,40,41]:

(7) $$\frac{t}{q_{t}}=\frac{1}{k_{2}q_{e}{}^{2}}+\frac{1}{q_{e}}t\;$$

where *k*_2_ (g/mg·min) denotes the adsorption rate constant of reactive dye, and *q_e_* and *q_t_* denote the masses of dye which react with cotton fabric samples at equilibrium state and at given times *t*, respectively.

As shown in Equation (7), *R*^2^ was calculated using plots of *t/q_t_* versus adsorption time (*t*).We can use the slopes and intercepts of these plots to calculate the adsorption rate of reactive dye (*k*_2_) and the adsorption equilibrium (*q_e_*), as shown in Equation (8):

(8) qe=1/tanαk2=1bqe2 

where tan *α* and *b* denote the slope and intercept of the linear equation, respectively.

The dyeing rate can also be expressed by the half-dyeing time (*t*_0.5_) which is the time required for half of the dye to reach equilibrium adsorption amount. The *t*_0.5_ was calculated using Equation (9):

(9) $$t_{0.5}=\frac{1}{k\cdot q_{e}}\;$$

**Figure 7:**
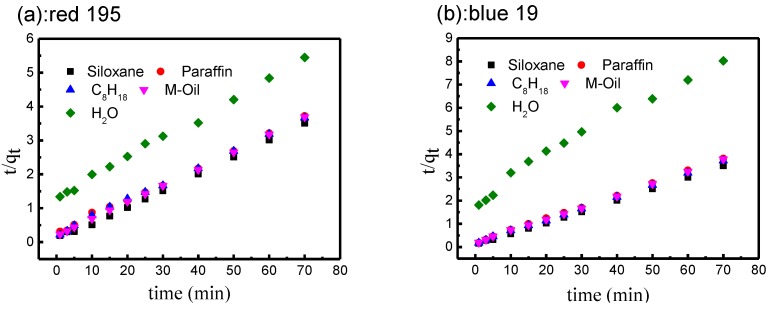
Kinetics of pseudo-second-order model for adsorption of reactive dyes: (**a**) red 195; (**b**) blue 19.

Figure 7 shows the fitting curves of the pseudo-second-order model and Table 3 lists the results of rate constant studies for different non-aqueous dyeing systems and the conventional water dyeing system with the pseudo-second-order model. Both non-aqueous dyeing systems and the aqueous system showed a high correlation coefficient value (>0.991). Furthermore, the equilibrium adsorption capacities, *q_e,cal_*, fit well with the experimental adsorption capacities, *q_e,exp_*, in the non-aqueous dyeing systems and the traditional aqueous system. These results suggest that the adsorption kinetics of these two reactive dyes on a cotton fabric surface can be described by the pseudo-second-order adsorption mechanism in non-aqueous dyeing systems and a traditional aqueous system.

The results in Table 3 also show *k*_2_, *t*_0.5_, as a main factor in the adsorption rate. For the adsorption rate, the rate constant in non-aqueous dyeing systems was significantly faster than that of the traditional water bath. Especially in the siloxane non-aqueous dyeing system, the rate constants of these two reactive dyes were 5~6 times those of the conventional aqueous bath. In addition, the half-dyeing time, *t_0.5_*, was significantly lower in non-aqueous dyeing systems than in the aqueous dyeing system. These results indicated that the adsorption of reactive dye in non-aqueous dyeing systems was faster than in the conventional water system.

Based on the amount of reactive dye (2%, o.w.f) added to the dyeing system, if all of the dye was adsorbed on the cotton fabric, the adsorption capacity was 20 mg/g. For reactive red 195, the equilibrium adsorption, *q_e,exp_*, at 10 min reached above 18.81 mg/g in the non-aqueous dyeing systems, indicating that at least 94.05% of dye had been adsorbed on the cotton fabric. However, only 13.75 mg/g (68.75%) of dye was adsorbed in the water bath after dyeing for 70 min. We also observed a similar phenomenon in the adsorption of reactive blue 19. These results suggest that the equilibrium time of reactive dye in non-aqueous dyeing systems was shorter than in the water bath, and almost all the dye could be adsorbed on the fabric in the non-aqueous dyeing systems. For different dyes, the rate constant value of red 195 was lower than that of blue 19 under the same dyeing conditions. The reason may be that the molecular structure of red 195 is complex and its molecular weight is higher than that of blue 19 [42,43,44], so the adsorption rate of the dye is influenced by the molecular structure.

#### 3.3.3. Effect of Non-Aqueous Media on the Dyeing Level Property

**Figure 8:**
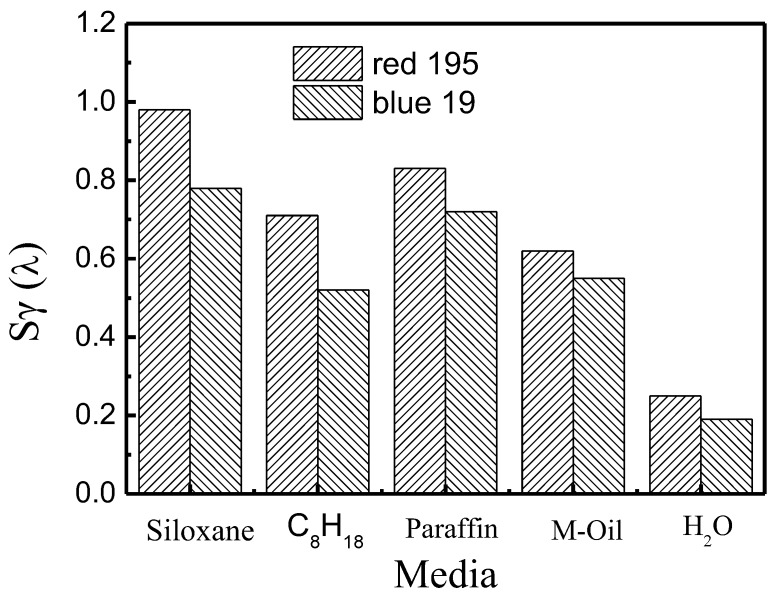
Level dyeing property of reactive dye in different non-aqueous dyeing systems and water bath.

**Figure 9:**
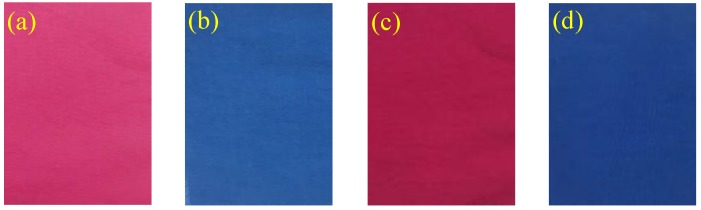
Level dyeing property of reactive dye in siloxane non-aqueous dyeing system and water bath: (**a**) red 195 in water base; (**b**) blue 19 in water base; (**c**) red 195 in siloxane non-aqueous dyeing system; (**d**) blue 19 in siloxane non-aqueous dyeing system.

Compared with a conventional aqueous bath, reactive dye has a faster adsorption rate in non-aqueous dyeing systems, which might influence their dyeing property. As shown in Figure 8 and Figure 9, for the dyeing property of reactive red 195 and blue 19, their values of *S_γ_*(*λ*) were higher in non-aqueous media than in the traditional water bath, but were less than 1, indicating that reactive dye can obtain a good level dyeing property by mechanical force during the dyeing process.

### 3.4. Influence of Non-Aqueous Media Surface Tension on Dye Adsorption Kinetics

#### 3.4.1. Non-Aqueous Media with Different Surface Tension

**Figure 10:**
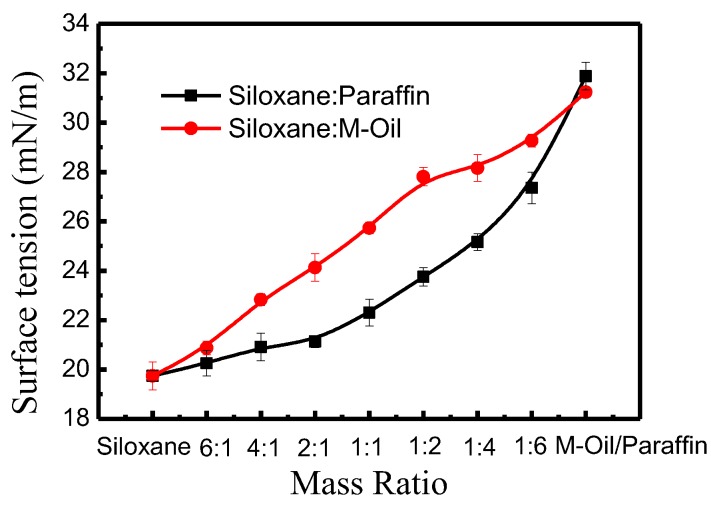
Different surface tensions of non-aqueous media after compounding siloxane with M-oil or paraffin.

Siloxane was formulated with M-oil or paraffin in a mass ration of 6:1, 4:1, 2:1, 1:1, 1:2, 1:4, and 1:6. Different surface tensions of the non-aqueous media are shown in Figure 10.

As shown in Figure 10, the surface tension of non-aqueous media increased with the increase of M-oil or paraffin. For example, when the mass ratio of siloxane to M-oil was 4:1, 1:1, and 1:4, the surface tensions of the mixed media were 22.83, 25.73, and 28.16 mN/m respectively. To study the influence of surface tension on reactive dye adsorption in non-aqueous dyeing systems, several mass ratios of siloxane to M-oil (4:1, 1:1, 1:4) were chosen in this investigation.

#### 3.4.2. Effect of Surface Tension on Reactive Dye Adsorption

**Figure 11:**
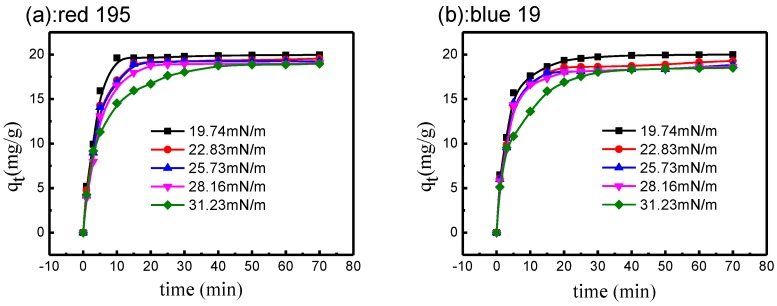
Adsorption rate of reactive dye in different surface tension non-aqueous dyeing media: (**a**) red 195; (**b**) blue 19.

As shown in Figure 11, in different surface tension non-aqueous dyeing systems, the uptake of reactive dyes was almost the same and was close to 100%, indicating that the surface tension of non-aqueous media had little influence on the final adsorption capacity of reactive dye. In the siloxane non-aqueous dyeing system, the reactive dye completes the adsorption process in 5–10 min. If M-oil was added to siloxane to increase the media surface tension, the adsorption rate of the reactive dye was decreased which meant it needed 10–15 min to reach the adsorption equilibrium. Moreover, reactive dye took more time (close to 20 min) to accomplish the adsorption process in M-oil non-aqueous dyeing media, in which the surface tension was 31.23 mN/m.

Thus, the above results reveal that, for dyeing with reactive dye in non-aqueous media, the higher surface tension of non-aqueous media is a possible reason for the longer adsorption time. This is due to the insolubility of reactive dye in non-aqueous media, and the strong affinity of reactive dye to cotton fabric [45,46]. Therefore, reactive dye can quickly diffuse to the fabric surface and complete the adsorption process [47,48,49]. When the surface tension of non-aqueous media is increased, the repulsive force between the reactive dye and non-aqueous media may decrease, which will influence the adsorption rate of the dye.

#### 3.4.3. Fitting of Pseudo-First-Order Kinetic Model

**Figure 12:**
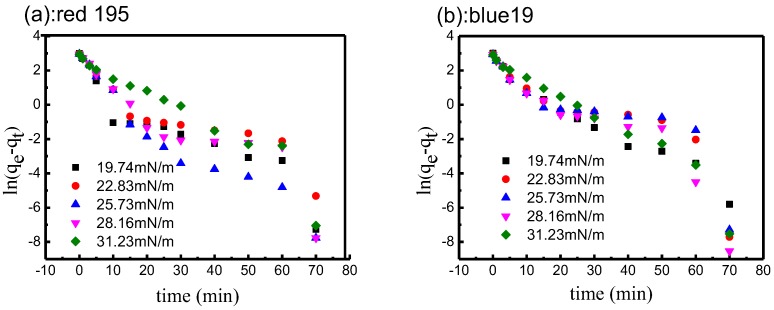
Kinetics of pseudo-first-order model for adsorption of reactive dyes in different surface tension non-aqueous dyeing systems: (**a**) red 195; (**b**) blue 19.

It can be seen from the results obtained in the above section, the salts can be eliminated and reactive dye can quickly diffuse to the cotton fabric surface in different surface tension non-aqueous dyeing processes. In order to thoroughly understand the effect of non-aqueous surface tension on the adsorption kinetics, the fitted line plots of equations for different surface tension non-aqueous dyeing media provide the values of *R^2^*. As shown in Figure 12 and Table 4, the correlation coefficients for different surface tension non-aqueous dyeing systems were above 0.744. However, the linearity was not good, indicating that the pseudo-first-order kinetic model did not fit well to the adsorption or reactive dye in different non-aqueous dyeing systems.

#### 3.4.4. Fitting of Pseudo-Second-Order Kinetic Model

**Figure 13:**
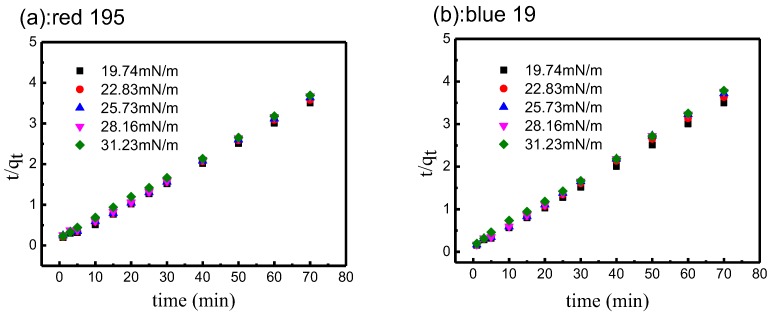
Kinetics of pseudo-second-order model for adsorption of reactive dyes in different surface tension non-aqueous dyeing systems: (**a**) red 195; (**b**) blue 19.

From the data in Table 5 and as shown in Figure 13, the plots provide higher correlation coefficients of more than 0.998, and excellent linearity for different surface tension non-aqueous dyeing systems. Moreover, the equilibrium adsorption capacities, *q_e,cal_*, fit well with the experimental adsorption capacities, *q_e,exp_*, in non-aqueous dyeing systems and in the traditional aqueous system. Therefore, the pseudo-second-order adsorption kinetic model was suitable to describe the adsorption kinetics of reactive dyes on cotton fabrics in different surface tension non-aqueous dyeing systems [50,51,52].

For the adsorption rate, the rate constant of reactive dye in lower surface tension non-aqueous dyeing systems was significantly faster than in higher surface tension systems. For example, the adsorption rate of reactive red 195 decreased from 3.18×10^−2^ to 2.11×10^−2^/(g/mg·min) when the non-aqueous surface tension increased from 19.74 to 28.16 mN/m. In addition, the half-dyeing time, *t*_0.5,_ was significantly lower in lower surface tension non-aqueous dyeing systems. These results indicated that the lower the surface tension of non-aqueous media, the faster was the adsorption rate of reactive dye, as well as the higher the final uptake of dye.

## 4. Conclusions

Reactive dyeing for cotton fabric and the adsorption kinetics of dye in non-aqueous dyeing systems, and a traditional water base, were investigated in this study. Salts were eliminated throughout the dyeing process because reactive dye was completely non-miscible with non-aqueous media, but had strong affinity to cotton fabric. Compared with reactive dyeing in a traditional water base, a higher (>94%) uptake of dye rendered the entire media recyclable. Moreover, the fixation of reactive dye was also higher (80%~90% for non-aqueous dyeing vs. 40%~50% for traditional dyeing), resulting in cotton fabric achieving a deeper shade after dyeing. The pseudo-second-order kinetic model can adequately describe the adsorption and equilibrium of reactive dyes in non-aqueous dyeing systems as well as in a traditional water dyeing system. In non-aqueous dyeing systems, the adsorption rate of reactive dye was significantly faster than in a traditional water bath. In the siloxane non-aqueous dyeing system, the adsorption equilibrium time of reactive dye was only 5–10 min at 25 °C, whereas it needed more time at 60 °C in the water dyeing system. The surface tension of non-aqueous media affected the adsorption rate of the dye. The lower the surface tension of non-aqueous media, the faster was the adsorption rate of reactive dye, and the higher the final uptake of dye. These favorable results have implications for reducing the environmental pressure from reactive dyeing for cotton textiles in a source-control manner through being salts-free, with higher dye uptake, lower dyeing temperature, and large water savings.

## Figures and Tables

**Table 1:** The parameters of different dyeing media.

Medium	Surface Tension (dyn/cm)	Boiling Point (°C)	Viscosity (mm^2^/s)
Siloxane	18.13	210	5.63
Paraffin	19.45	330	16.34
C_8_H_18_	23.34	126	0.77
M-Oil	31.23	200	4.20
H_2_O	72.04	100	1.00

**Table 2:** *R*^2^ of pseudo-first-order model for cotton fabric in non-aqueous dyeing media and water system.

Dye	Media				
	Siloxane	Paraffin	C_8_H_18_	M-Oil	H_2_O
Red-195	0.757	0.819	0.825	0.916	0.696
Blue-19	0.961	0.830	0.929	0.942	0.655

**Table 3:** Kinetic parameters of pseudo-second-order for adsorption of reactive red 195 and blue 19 in non-aqueous dyeing systems and traditional water bath.

Medium Dye	Parameter	Siloxane	Paraffin	C_8_H_18_	M-Oil	H_2_O
Red-195	*k*_2_×10^−2^ (g/mg·min)^−2^(min^−1^)	2.88	1.08	1.16	1.52	0.46
	*q_e,exp_* (mg/g)	19.97	18.81	19.08	18.97	12.86
	*q_e,cal_* (mg/g)	20.59	20.47	20.37	10.11	14.61
	*t*_0.5_ (min)	1.57	4.94	4.52	3.48	17.02
	*R*^2^	0.999	0.998	0.998	0.999	0.997
Blue-19	*k*_2_×10^−2^ (g/mg·min)^−2^(min^−1^)	3.18	1.62	1.82	1.74	0.63
	*q_e,exp_* (mg/g)	19.99	18.35	18.83	18.50	8.72
	*q_e,cal_* (mg/g)	20.64	19.33	19.80	19.54	11.30
	*t*_0.5_ (min)	1.74	3.38	2.92	3.11	18.19
	*R*^2^	0.999	0.999	0.999	0.999	0.991

**Table 4:** *R*^2^ of pseudo-first-order model for cotton fabric in different surface tension non-aqueous dyeing system.

Dye	Siloxane:W-Oil	*R*^2^
Reactive red 195	1:0	0.852
	4:1	0.847
	1:1	0.918
	1:4	0.838
	0:1	0.916
Reactive blue 19	1:0	0.962
	4:1	0.782
	1:1	0.744
	1:4	0.868
	0:1	0.942

**Table 5:** *R*^2^ of pseudo-second-order model for cotton fabric in different surface tension non-aqueous dyeing systems.

Dye	Surface Tension (mN/m)	*q_e,exp_* (g/mg)	Second-Order Kinetic Model			
			*k*_2_×10^−2^ (g/mg·min)	*q_e,cal_* (mg/g)	*t*_0.5_ (min)	*R*^2^
Red 195	19.74	19.97	3.18	20.59	1.57	0.998
	22.83	19.55	2.47	20.19	2.07	0.999
	25.73	19.26	2.29	20.11	2.27	0.998
	28.16	19.06	2.11	20.00	2.49	0.998
	31.23	18.97	1.52	20.11	3.48	0.999
Blue19	19.74	19.98	2.88	20.64	1.74	0.999
	22.83	19.28	2.62	19.82	1.98	0.999
	25.73	18.83	2.24	19.25	2.37	0.999
	28.16	18.61	2.14	19.19	2.51	0.999
	31.23	18.50	1.74	19.54	3.11	0.999

## References

[1] Almasian A., Olya M.E., Mahmoodi N.M. (2015). Synthesis of polyacrylonitrile/polyamidoamine composite nanofibers using electrospinning technique and their dye removal capacity. J. Taiwan Inst. Chem. Eng..

[2] Mao X., Zhong Y., Xu H., Zhang L., Sui X., Mao Z. (2018). A novel low add-on technology of dyeing cotton fabric with reactive dyestuff. Text. Res. J..

[3] Ali S., Khatri Z., Khatri A., Tanwari A. (2014). Integrated desizing-bleaching-reactive dyeing process for cotton towel using glucose oxidase enzyme. J. Clean. Prod..

[4] Banchero M. (2013). Supercritical fluid dyeing of synthetic and natural textiles—A review. Color. Technol..

[5] Cid M.V.F., Spronsen J.V., Kraan M.V.D., Veugeler W.J.T., Woerlee G.F., Witkamp G.J. (2007). A significant approach to dye cotton in supercritical carbon dioxide with fluorotriazine reactive dyes. J. Supercrit. Fluid..

[6] Tang Y.L., Lee C.H., Wang Y., Kan C.W. (2017). Octane-assisted reverse micellar dyeing of cotton with reactive dyes. Polymers.

[7] Banchero M., Ferri A. (2005). Simulation of aqueous and supercritical fluid dyeing of a spool of yarn. J. Supercrit. Fluid..

[8] Fang K., Shu D., Liu X., Cai Y., An F., Zhang X. (2018). Reactive pad-steam dyeing of cotton fabric modified with cationic p(st-ba-vbt) nanospheres. Polymers.

[9] Chen L., Wang B., Ruan X., Chen J., Yang Y. (2015). Hydrolysis-free and fully recyclable reactive dyeing of cotton in green, non-nucleophilic solvents for a sustainable textile industry. J. Clean. Prod..

[10] Xie K., Cheng F., Zhao W., Xu L. (2011). Micelle dyeing with low liquor ratio for reactive dyes using dialkyl maleic acid ester surfactants. J. Clean. Prod..

[11] Radei S.F., Javier C.F., Ardanuy M., José M.C. (2018). Kinetics of low temperature polyester dyeing with high molecular weight disperse dyes by solvent microemulsion and agrosourced auxiliaries. Polymers.

[12] Yi S., Dong Y., Li B., Ding Z., Huang X., Xue L. (2012). Adsorption and fixation behaviour of ci reactive red 195 on cotton woven fabric in a nonionic surfactant triton X-100 reverse micelle. Colo. Technol..

[13] Xu C., Zhang L., Xu D., Li M., Zhang Y., Fu S. (2017). Preparation of reactive nanoscale carbon black dispersion for pad coloration of cotton fabric. Color. Technol..

[14] Wang B., Ruan X., Chen L., Chen J., Yang Y. (2015). Heterogeneous chemical modification of cotton cellulose with vinyl sulfone dyes in non-nucleophilic organic solvents. Ind. Eng. Chem. Res..

[15] Fu C., Wang J., Shao J., Pu D., Chen J., Liu J. (2015). A non-aqueous dyeing process of reactive dye on cotton. J. Tex. Inst..

[16] Pei L., Liu J., Cai G., Wang J. (2017). Study of hydrolytic kinetics of vinyl sulfone reactive dye in siloxane reverse micro-emulsion. Text. Res. J..

[17] Pei L., Liu J., Wang J. (2017). Study of Dichlorotriazine Reactive Dye Hydrolysis in Siloxane Reverse Micro-emulsion. J. Clean. Prod..

[18] Pei L., Wu P., Liu J., Wang J. (2017). Effect of nonionic surfactant on the micro-emulsifying water in silicone media. J. Surfactants Deterg..

[19] Miao H., Liu J., Li Y., Fu C., Zhang Y. (2013). Study on hydrolysis kinetics of reactive dyes in dye/D5 suspending system. J. Tex. Res..

[20] Burns-Naas L.A., Mast R.W., Meeks R.G., Mann P.C., Thevenaz P. (1998). Inhalation toxicology of decamethylcyclopentasiloxane (D5) following a 3-monthnose-only exposure in Fischer 344 rats. Toxicol. Sci..

[21] Reddy M.B., Looney R.J., Utell M.J., Plotzke K.P., Andersen M.Z. (2007). Modeling of human dermalabsorption of octamethylcyclotetrasiloxane (D4) anddecamethylcyclopentasiloxane (D5). Toxicol. Sci..

[22] Jovanovic M.L., McMahon J.M., McNett D.A., Tobin J.M., Plotzke K.P. (2008). In vitro and in vivo percutaneousabsorption of 14 C-octamethylcyclotetrasiloxane (14C-D4) and 14C-decamethylcyclopentasiloxane (14C-D5). Regul. Toxicol.Pharmacol..

[23] Gao X.Y., Liu J.Q., Li Y.Q. (2007). The impact of D5-washing on the fabric performance. J. Zhejiang Sci.-Tech. Univ..

[24] Ahmed N.S.E. (2005). The use of sodium edate in the dyeing of cotton with reactive dyes. Dyes Pigment..

[25] Gök Ö., Özcan A.S., Özcan A. (2010). Adsorption behavior of a textile dye of Reactive Blue 19 from aqueous solutions onto modified bentonite. Appl. Surf. Sci..

[26] Gong J., Ren Y., Fu R., Li Z., Zhang J. (2017). Ph-mediated antibacterial dyeing of cotton with prodigiosins nanomicelles produced by microbial fermentation. Polymers.

[27] Renfrew A.H.M. (2010). Reactive Dyes for Cellulose: Replacement of Anthraquinone Blues by Triphenodioxazines. Color. Technol..

[28] Al-Degs Y.S., El-Barghouthi M.I., El-Sheikh A.H., Walker G.M. (2008). Effect of solution pH, ionic strength, and temperature on adsorption behavior of reactive dyes on activated carbon. Dyes Pigment..

[29] Chiou M.S., Li H.Y. (2003). Adsorption behavior of reactive dye in aqueous solution on chemical cross-linked chitosan beads. Chemosphere.

[30] Vanaamudan A., Sudhakar P.P. (2015). Equilibrium, kinetics and thermodynamic study on adsorption of reactive blue-21 and reactive red-141 by chitosan-organically modified nanoclay (Cloisite 30B) nano-bio composite. Taiwan Inst. Chem. Eng..

[31] Bhavsar P.S., Zoccola M., Patrucco A., Montarsolo A., Mossotti R., Giansetti M., Rovero G., Maier S.S., Muresan A., Tonin C. (2017). Superheated water hydrolyzed keratin: A new application as a foaming agent in foam dyeing of cotton and wool fabrics. ACS Sustain. Chem. Eng..

[32] Annadurai G., Ling L.Y., Lee J.F. (2008). Adsorption of reactive dye from an aqueous solution by chitosan: Isotherm, kinetic and thermodynamic analysis. J. Hazard. Mater..

[33] Sawada K., Ueda M. (2003). Dyeing of protein fiber in a reverse micellar system. Dyes Pigment..

[34] Ho Y.S., McKay G. (1999). Comparative sorption kinetic studies of dyes and aromatic compounds onto flyash. J. Environ. Sci. Health. Part A.

[35] Ma Q.Y., Wang L.J. (2015). Adsorption of Reactive blue 21 onto functionalized cellulose under ultrasonic pretreatment: Kinetic and equilibrium study. J. Taiwan Inst. Chem. Eng..

[36] Demir H., Top A., Balköse D., Ulkü S. (2008). Dye adsorption behavior of luffacylindrica fibers. J. Hazard. Mater..

[37] Liu C., Chan K., Shen J., Liao C., Yeung K., Tjong S. (2016). Polyetheretherketone hybrid composites with bioactive nanohydroxyapatite and multiwalled carbon nanotube fillers. Polymers.

[38] Blanco S.P.D.M., Scheufele F.B., Módenes A.N., Espinoza-Quiñones F.R., Marin P., Kroumov A.D., Borba C.E. (2016). Kinetic, equilibrium and thermodynamic phenomenological modeling of reactive dye adsorption onto polymeric adsorbent. Chem. Eng. J..

[39] Ho Y.S., McKay G. (2000). The kinetics of sorption of divalent metal ions onto sphagnum moss peat. Water Res..

[40] Wang X., Huang K., Chen Y., Liu J., Chen S., Cao J., Mei S., Zhou Y., Jing T. (2018). Preparation of dumbbell manganese dioxide/gelatin composites and their application in the removal of lead and cadmium ions. J. Hazard. Mater..

[41] Wu F.C., Tseng R.L., Juang R.S. (2001). Kinetic modeling of liquid-phase adsorption of reactive dyes and metal ions on chitosan. Water Res..

[42] Arslan-Alaton I. (2003). The effect of pre-ozonation on the biocompatibility of reactive dye hydrolysates. Chemosphere.

[43] Mane V.S., Mall I.D., Srivastava V.C. (2007). Kinetic and equilibrium isotherm studies for the adsorptive removal of Brilliant Green dye from aqueous solution by rice husk ash. J. Environ. Mang..

[44] Morris K.F., Lewis D.M., Broadbent P.J. (2008). Design and application of a multifunctional reactive dye capable of high fixation efficiency on cellulose. Color. Technol..

[45] Duan Y., Liu Y., Li J., Wang H., Wen S. (2018). Investigation on the nanomechanics of liposome adsorption on titanium alloys: Temperature and loading effects. Polymers.

[46] McKay G., Otterburn M.S., Sweeney A.G. (1980). The removal of color from effluent using various adsorbents III. Silica: Rate processes. Water Res..

[47] Silva T.L., Ronix A., Pezoti O., Souza L.S., Leandro P.K.T., Bedin K.C., Beltrame K.K., Cazetta A.L., Almeida V.C. (2016). Mesoporous activated carbon from industrial laundry sewage sludge: Adsorption studies of reactive dye remazol brilliant blue. Chem. Eng. J..

[48] Wang L., Li J., Adsorption of C.I. (2013). Reactive Red 228 dye from aqueous solution by modified cellulose from flax shive: Kinetics, equilibrium, an thermodynamics. Ind. Crop. Prod..

[49] Marin P., Borba C.E., Módenes A.N., Espinoza-Quiñones F.R., Oliveira S.P.D.D., Kroumov A.D. (2014). Determination of the mass transfer limiting step of dye adsorption onto commercial adsorbent by using mathematical models. Environ. Technol..

[50] Moradi O., Gupta V.K., Agarwal S., Tyagi I., Asif M., Makhlouf A.S.H., Sadegh H. (2015). Characteristics and electrical conductivity of graphene and graphene oxide for adsorption of cationic dyes from liquids: Kinetic and thermodynamic study. J. Ind. Eng. Chem..

[51] Hasan M., Ahmad A.L., Hameed B.H. (2008). Adsorption of reactive dye onto cross-linked chitosan/oil palm ash composite beads. Chem. Eng. J..

[52] Castaldo R., Gentile G., Avella M., Carfagna C., Ambrogi V. (2017). Microporous hyper-crosslinked polystyrenes and nanocomposites with high adsorption properties: A review. Polymers.

